# The Association between Nutritional Status and In-Hospital Mortality among Patients with Heart Failure—A Result of the Retrospective Nutritional Status Heart Study 2 (NSHS2)

**DOI:** 10.3390/nu13051669

**Published:** 2021-05-14

**Authors:** Michał Czapla, Raúl Juárez-Vela, Katarzyna Łokieć, Piotr Karniej

**Affiliations:** 1 Faculty of Health Sciences, Wroclaw Medical University, 51-618 Wroclaw, Poland; michal.czapla@umed.wroc.pl (M.C.); piotr.karniej@umed.wroc.pl (P.K.); 2 Centre for Heart Diseases, University Hospital, 50-566 Wroclaw, Poland; 3 Faculty of Medicine, University of Salamanca, 37008 Salamanca, Spain; 4 Department of Propaedeutic of Civilization Diseases, Medical University of Lodz, 90-251 Lodz, Poland; katarzyna.lokiec@umed.lodz.pl

**Keywords:** malnutrition, obesity paradox, heart failure, nutritional status, NRS2002, lipid paradox

## Abstract

Background: A nutritional status is related to the prognosis and length of hospitalisation of patients with heart failure (HF). This study aims to assess the effect of nutritional status on in-hospital mortality in patients with heart failure. Methods: We conducted a retrospective study and analysis of medical records of 1056 patients admitted to the cardiology department of the University Clinical Hospital in Wroclaw (Poland). Results: A total of 1056 individuals were included in the analysis. A total of 5.5% of patients died during an in-hospital stay. It was found that in the sample group, 25% of patients who died had a BMI (body mass index) within the normal range, 6% were underweight, 47% were overweight, and 22% were obese. Our results show that non-survivors have a significantly higher nutrition risk screening (NRS) ≥3 (21% vs. 3%; *p* < 0.001); NYHA (New York Heart Association) grade 4 (70% vs. 24%; *p* < 0.001). The risk of death was lower in obese patients (HR = 0.51; *p* = 0.028) and those with LDL (low-density lipoprotein) levels from 116 to <190 mg/dL (HR = 0.10; *p* = 0.009, compared to those with LDL <55 mg/dL). The risk of death was higher in those with NRS (nutritional risk score) score ≥3 (HR = 2.31; *p* = 0.014), HFmrEF fraction (HR = 4.69; *p* < 0.001), and LDL levels > 190 mg/dL (HR = 3.20; *p* = 0.038). Conclusion: The malnutrition status correlates with an increased risk of death during hospitalisation. Higher TC (total cholesterol) level were related to a lower risk of death, which may indicate the “lipid paradox”. Higher BMI results were related to a lower risk of death, which may indicate the “obesity paradox”.

## 1. Introduction

In 2015, one-third of all deaths have been caused by cardiovascular diseases (CVDs), which are among the most common causes of death not only in Europe but globally [1,2]. Heart failure (HF) is one of them. It has a high incidence rate, high hospitalisation rate, and significant mortality rate [3]. In many countries, HF is the major cause of death in the elderly, which is an important and frequently underestimated public health problem [4]. Lifestyle changes in CVD prevention, regardless of the patients’ age, are of great importance, and they are estimated to prevent approximately 80% of CVD cases [5]. However, an interdisciplinary approach and collaboration among physicians, nurses, dietitians, physiotherapists, public health assistants, and other medical professionals are required for such changes to be effective. There is no doubt that nutritional status and HF are interrelated. Malnutrition worsens patients’ quality of life [6]. It also increases hospitalisation rates and mortality rates [7]. The problem of malnutrition is estimated to affect approximately 30% of all hospitalised patients; screening assessment tools (SATs) are used for its identification. The nutritional risk screening (NRS2020) is one of the SATs recommended by the European Society of Parenteral Enteral Nutrition (ESPEN) [8]. Each patient admitted to the hospital should undergo a nutritional assessment. HF may be caused by low nutrient intake in the setting of intestinal oedema, malabsorption, or increased basal metabolic rate. This leads to malnutrition and means not only worse acceptance of illness but also decreased responsiveness to the pharmacotherapy used [9]. Another measure that can be used for assessing malnutrition is body mass index (BMI). Here, the results are related to the prognosis of HF patients. BMI itself does not necessarily indicate malnutrition, due to possible fluid retention which is common in HF patients [10].

The HF patients must undergo a multi-stage and long treatment process. In the clinical setting, the process starts with the patient’s admission to the hospital and ends with the changes in the patient’s eating habits, intensive cardiac rehabilitation, implantation of an electrostimulation device, sometimes even a heart transplant. It should also be noted that chronic conditions accompanying HF and malnutrition affect the course of the disease [11].

This study aims to assess how nutritional status affects in-hospital mortality in patients with heart failure.

## 2. Materials and Methods

### 2.1. Study Design and Setting

A retrospective study and analysis of medical records of patients admitted to the cardiology department of the University Clinical Hospital in Wroclaw (Poland) between September 2017 and September 2020 due to acute heart failure (HF) (ICD10:I50) were carried out. STROBE (Strengthening the Reporting of Observational Studies in Epidemiology) guidelines were followed.

### 2.2. Study Population

We analysed all the patients who met the inclusion criteria (diagnosis of HF, age ≥18 years old). The medical records of a final group of 1056 patients were analysed. The analysis included data such as patients’ age, sex, body mass index (BMI), and laboratory results such as total cholesterol (TC), high-density lipoprotein (HDL), low-density lipoprotein (LDL), triglycerides (TG), HF type, New York Heart Association (NYHA) classification, data concerning past and comorbid disease entities, and assessment of the nutritional status of the patient using NRS-2002.

### 2.3. Nutritional Screening

NRS-2002 is one of the tools for the screening assessment of nutritional status, recommended by the Global Leadership Initiative on Malnutrition (GLIM) [12]. The aforementioned scale consists of two stages: 1. Impaired nutritional status, in which weight loss in the period of up to three months and BMI (body mass index) are assessed. The same applies to the percentage of food intake compared to food requirements within the last week. In this stage, the patient may achieve 0–3 points, where 0 points mean no health deterioration and 3 points mean severe health deterioration. 2. Severity of disease (an increase in requirements), where, depending on the disease, patients may obtain 0–3 points, where 0 points mean normal nutritional requirements and 3 points mean high disease severity (e.g., marrow transplant, patient in an intensive care unit). Moreover, patients aged over 70 receive 1 additional point. Patients can score 0–7 points. Nutritional therapy is recommended in patients with NRS ≥ 3 [12,13]]. The WHO criteria were used for the classification of patients as underweight (BMI < 18.5), with normal weight (BMI 18.5–24.9), pre-obese (BMI 25–29.9), and obese (BMI ≥ 30).

Both NRS-2002 and BMI were calculated by the physician admitting the patient to the department of cardiology.

### 2.4. Data Collection

The analysis involved both categorical variables and continuous variables. The categorical variables include sex, BMI (18.5–24.9, <18.5, 25.0–29.9, ≥30), NRS (<3 vs. ≥3), heart failure phenotype (HFpEF, HFmrEF, HFrEF, no information), NYHA (1,2,3,4), MI (Yes/No), ventricular fibrillation (Yes/No), CKD (Yes/No), type of MI (No, STEMI, NSTEMI, no information), hypertension (Yes/No), diabetes mellitus (Yes/No), cerebral stroke (Yes/No), thyroid disease (normal, hyperthyroidism, hypothyroidism), LDL (<55 mg/dL, 55 to <70 mg/dL, 70 to <100 mg/dL, 100 to <116 mg/dL, 116 to <190 mg/dL, more than 190 mg/dL), LDL (≥70 vs. <70), TG (<135, 135–200, >200), HDL (<40, >40). The continuous variables include age, BMI, height [m], body weight [kg], NT-proBNP pg/mL, BNP [pg/mL], triglycerides (TG) [mg/dL], LDL cholesterol [mg/dL], HDL cholesterol [mg/dL], total cholesterol [mg/dL], ultra-sensitive CRP [mg/L], albumen [g/dL], transferrin [g/L], lymphocytes [%], procalcitonin, PCT [ng/mL]. The variables such as BMI and LDL were analysed in the univariate model as continuous and categorical variables. In the case of the final multivariate model, the variables (BMI and LDL) were selected depending on the better fit of the model based on the assessment of the goodness of fit (GOF).

### 2.5. Statistical Analysis

The statistical analysis was performed using Statistica 13.1 software (TIBCO, Inc., Palo Alto, CA, USA). Arithmetic means and standard deviations were calculated for measurable variables. Quantitative variables were tested using the Shapiro–Wilk test to determine the distribution type. Intergroup comparisons were performed using the *t*-test or Mann–Whitney U test (depending on whether the assumptions were met). The comparison of results of more than two groups was performed using the one-way analysis of variance (ANOVA) or the Kruskal–Wallis test (depending on whether the assumptions were met). The survival analysis was performed using the Kaplan–Meier method. The log-rank test was used to compare patient survival against selected clinical variables. The Cox proportional hazards model was used for assessing the influence of qualitative or quantitative variables on patient survival. The results were considered statistically significant at *p* < 0.05. The model-building process was conducted using a progressive stepwise method and a set of standard measures of the goodness of fit (AIC, R2) was used for assessing the model. The results were considered statistically significant at *p* < 0.05.

## 3. Results

### 3.1. Characteristics of the Group

The profile of the whole group with a comparison of characteristics between the group of survivors and non-survivors is shown in Table 1. A total of 1056 individuals were included in the analysis. Due to a lack of data for some parameters, those numbers are smaller and are provided for each variable. Patients with a BMI between 25.0 and 29.9 were statistically significantly more likely to die (47%, *n* = 17). The non-survivors had higher parameters such as NRS score above ≥3 (21% vs. 3%; *p* < 0.001); NYHA grade 4 (70% vs. 24 %; *p* < 0.001). Statistically significant differences were also observed for the prevalence of different LDL levels. A significantly higher percentage of survivors had HDL levels higher than 40 (49% vs. 25%, *p* = 0.001; Table 1). Furthermore, non-survivors were older ($\overset{–}{X}$ = 77.7 vs. $\overset{–}{X}$ = 69.3 years of age; *p* < 0.001) and had lower body weight ($\overset{–}{X}$ = 75.5 vs. $\overset{–}{X}$ = 83.3 kg; *p* = 0.019). In the group of non-survivors, considering the laboratory parameters, a statistically significantly higher result was obtained in the levels of BNP, ultra-sensitive CRP, and procalcitonin. In non-survivors, lower scores were observed in the assessment of parameters such as LDL, total cholesterol, albumin, transferrin, or lymphocytes (Table 1).

### 3.2. Subgroup Analysis According to BMI

A comparison of the assessed variables according to BMI is shown in Table 2. Based on BMI, four groups were distinguished: <18.5, 18.5–24.9, 25.0–29.9, and ≥30. Statistically significant differences were found when considering sex, NRS, hypertension, diabetes mellitus (DM), TG, HDL. Male patients constituted a higher percentage in the group with BMI ranges of 18.5–24.9, 25.0–29.9, and above 29.9. The occurrence of DM was significantly more frequently observed in the group of obese patients (58%, *n* = 171). The same applies to hypertension (80.7%, *n* = 239; Table 2). Additionally, lower age, NT-proBNP, BNP, LDL, HDL, and TC were observed in the group with BMI ≥30 (Table 3).

### 3.3. Subgroup Analysis According to NRS

A comparison of the assessed parameters according to NRS scores is shown in Table 4. Based on the NRS score, two groups were distinguished: NRS <3 and NRS ≥3. Statistically significant differences were found for BMI, HF phenotype, NYHA, or patients with diabetes. A significantly more frequently impaired nutritional status was observed in patients with normal BMI. DM was more common in patients with NRS <3. Of patients with NRS ≥3, 50% presented in NYHA class 4 (Table 4).

The group with NRS ≥3 had a mean higher age, BNP, and CRP levels than the group with NRS <3 (*p* < 0.001). Additionally, lower scores of BMI, body height, body weight, LDL, cholesterol, HDL, and lymphocytes were observed in the group with NRS ≥3 (Table 5).

### 3.4. Survival Analysis

**Figure 1:**
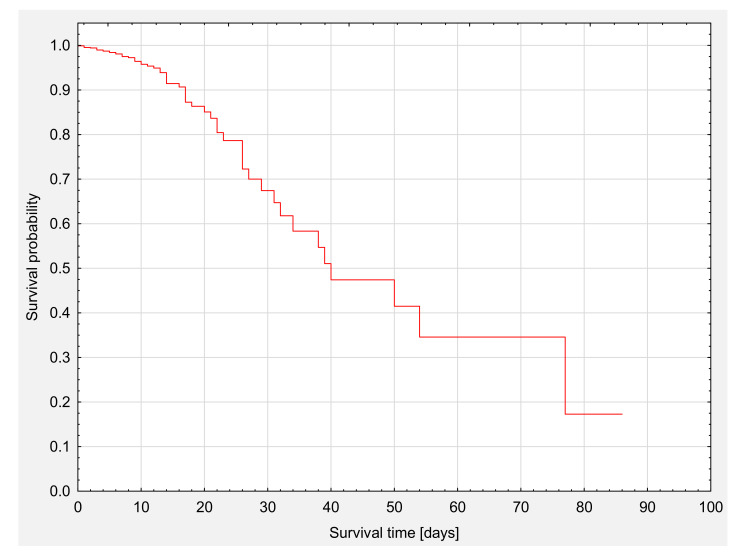
The analysis of survival of the whole group.

The profile of the whole group of patients’ survival analysis is presented on Kaplan–Meier survival curves (Figure 1). The median survival was 39 days (Table 6). The total survival rate was 94.5% (*n* = 998).

### 3.5. Survival Analysis—Group Comparisons

**Figure 2:**
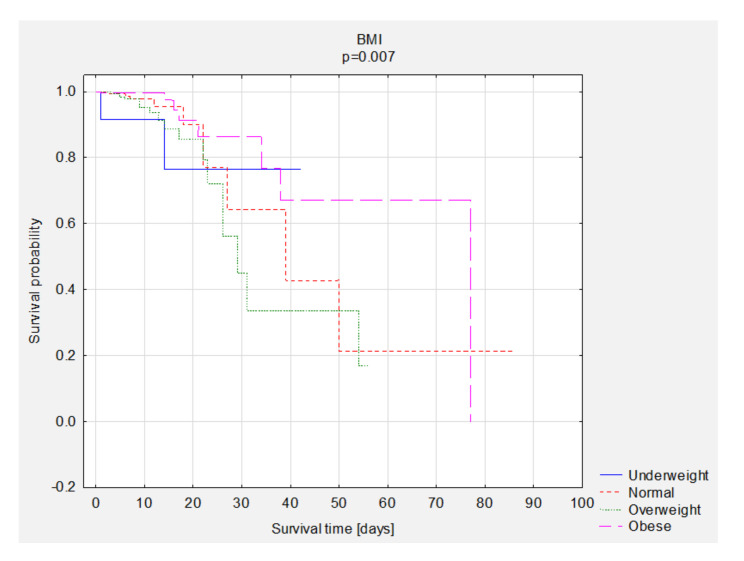
The comparison of survival curves according to BMI results. Abbreviations: BMI, body mass index.

**Figure 3:**
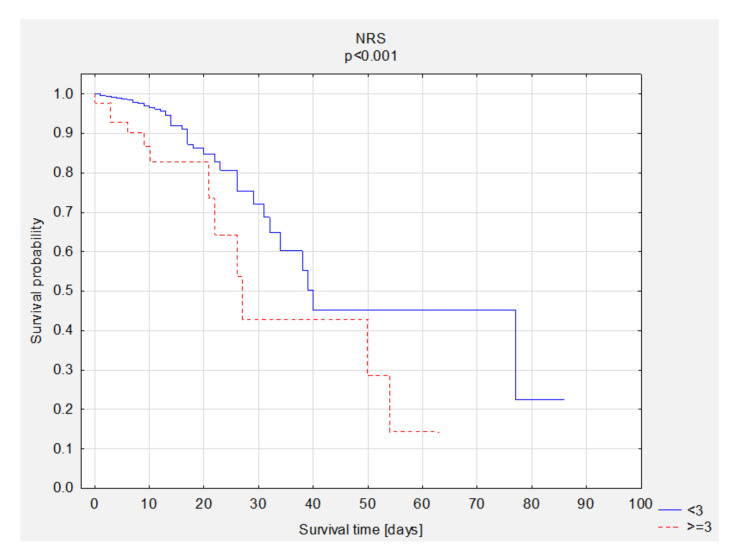
The comparison of survival curves according to NRS scores. Abbreviations: NRS, nutritional risk screening.

**Figure 4:**
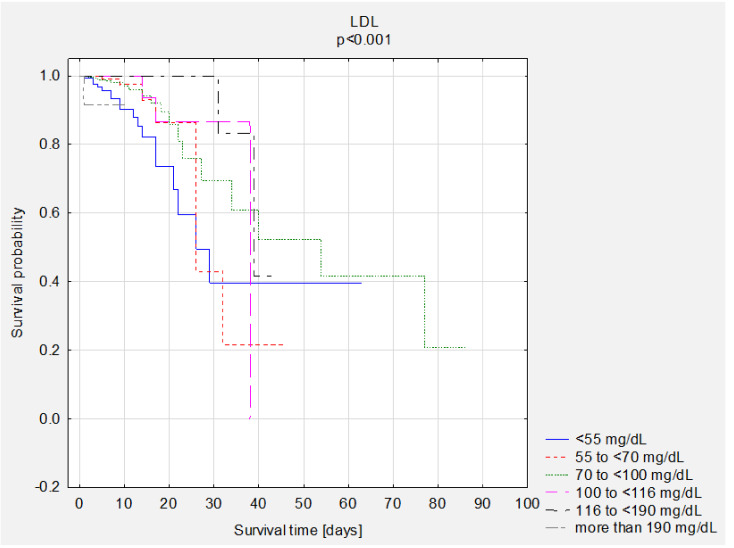
The comparison of survival curves according to LDL scores. Abbreviations: LDL, low-density lipoprotein.

A comparison of survival curves according to BMI, NRS, and LDL was performed. Statistically significant better survival of obese patients was observed (Figure 2). The overall survival rate was 83% in the group of underweight patients, 96% in the group of patients with normal BMI, 94% in the group of overweight patients, and 97% in the group of obese patients. A better survival rate was observed in patients with better nutritional status (Figure 3). A comparison of survival curves according to LDL levels was performed (Figure 4). The overall survival rate was 95% in the group with NRS <3 and 73% in the group with NRS ≥3. The best survival percentage was observed in the group with LDL levels of 116 to <190 mg/dL—99%, followed by 100 to <116 mg/dL—98%, 55 to ≤70 mg/dL—96%, 70 ≤100 mg/dL—95%, >190 mg/dL—91%, and <55 mg/dL—88% (Figure 4). We performed descriptive statistics for survival time and number of deaths and survival depending on the BMI results, NRS scores and LDL scores (Table 7). 

The assessment of the influence of selected variables on mortality is shown in Table 8 (Cox proportional hazards regression). It was observed that the risk of death in obese patients (HR = 0.51; *p* = 0.028) and those with LDL levels between 116 and <190 mg/dL (HR = 0.10; *p* = 0.009, compared to those with LDL <55 mg/dL) decreased. In contrast, there was an increase in the risk of death when NRS scores were equal to or greater than 3 (HR = 2.31; *p* = 0.014), the HF phenotype was HFmrEF (HR = 4.69; *p* < 0.001, compared to HFpEF), and the LDL levels were greater than 190 mg/dL (HR = 3.20; *p* = 0.038).

Considering the quantitative variables, there was a reduced risk of death when patients achieved higher BMI results, as well as LDL, HDL, total cholesterol, albumin, and lymphocyte scores. Mortality was influenced by higher scores in the parameters such as age, BNP, triglycerides, CRP, and procalcitonin.

Variables were included in the multivariate model in accordance with the adopted criteria. Those criteria included the outcome of *p* < 0.30 in a univariate model, a lack of correlation of variables, as well as clinical recommendations. The variables included in the model were as follows: HDL, CRP, total cholesterol, sex, HF phenotype, NRS, BMI, NYHA, LDL, TG. The multivariate analysis showed that higher total cholesterol levels (HR = −0.02; *p* = 0.005) and obesity (HR = 0.20; *p* = 0.004) correlate with mortality (Table 9).

## 4. Discussion

The malnutrition status in HF patients is undoubtedly related to the quality of life, risk of re-hospitalisation, prolonged hospitalisation, complications, and increased risk of death during in-patient treatment [14]. Consequently, disease-related malnutrition significantly increases medical costs, and its importance in the course of the disease remains underappreciated [15,16]. In this study, using the univariate analysis, the risk of malnutrition more than doubled the risk of death in HF patients (HR = 2.31; *p* = 0.014). As regards the group of patients diagnosed with malnutrition, the rate of mortality during hospitalisation was 27%. It was also observed that the risk of death increased with age (HR = 1.05; *p* < 0.001). A similar result in HF patients was obtained by Azzis et al. In their study, the risk of malnutrition was related to a threefold increase in the risk of all-cause mortality in HF patients [17]. In the case of patients with other CVDs, the situation is similar. According to Ya-Wen Lu et al.’s study, the rate of in-hospital mortality in malnourished patients was less than 20%. The authors also found that the risk of death increased with age (HR 1.04 *p* = 0.002) and was 3.5 times higher (HR 3.47 *p* < 0.001) in patients diagnosed with malnutrition [18]. Also, in the study by Ponilla-Palomas et al., the risk of death was four times higher in patients diagnosed with malnutrition [11]. This is why early diagnosis of malnutrition and appropriate nutritional intervention are of great importance.

According to the observations of the authors of this study, in the group of patients with LDL scores of 116 to <190 mg/dL, 99% of patients survived. In the group of patients with LDL scores of <55 mg/dL, however, 88% of patients survived. The risk of death during hospitalisation was lower in patients with LDL between 116 and <190 mg/dL (HR = 0.10; *p* = 0.009 compared to those with LDL <55 mg/dL). In contrast, the risk of death during hospitalisation increased when LDL levels were greater than 190 mg/dL (HR = 3.20; *p* = 0.038). In the case of primary prevention of CVDs in high-risk patients, ESC recommendations for LDL levels are <70 mg/dL [19]. The lipid paradox was reported in many clinical trials. Similar conclusions were reached by Charach et al., who also reported better survival in HF patients with higher LDL levels > 115 mg/dL. Those patients were slightly younger, and the prevalence of insulin-dependent diabetes, hypertension, and ischaemic heart disease was lower in that group [20]. That phenomenon was observed by researchers in other CVDs as well. Kyung Hoon Cho et al. studied a group of 9751 patients with acute coronary syndrome (ACS), who underwent percutaneous coronary intervention. In-hospital mortality was statistically significantly higher in patients with LDL >70 mg/dL. Depending on the model, the risk of death within 12 months from surgery was lower in patients with LDL of 70–99 than in those with LDL <70 (HR: 1.42 vs. 2.81). In this case, as in the presented study, the highest LDL levels (>160 mg/dL) no longer correlated with a lower risk of death, but with a higher risk. The authors point out that patients with low cholesterol levels were older and had more comorbidities [21]. Rauchhaus et al. also found that higher cholesterol levels may be related to better survival in HF patients. The mechanism of this paradox is not clear [22]. The lipid paradox was also confirmed in one of the largest cohort studies conducted in the United States by Vanessa et al., where it was found that a decrease in LDL levels were correlated with an increased risk of death during hospitalisation [23]. Also, higher TC levels were correlated with lower mortality risk. Similar results were obtained by Cuchna et al. [24]. Hence, the cause of the lipid paradox is not fully understood. Patients with heart failure usually suffer from multiple comorbidities. Therefore, the studied patients might have been treated with lipid-lowering drugs. Hence, low LDL levels may also be related to poor nutritional status. In the multivariate analysis, higher total cholesterol levels were significantly related to a lower risk of death. Such a relationship is confirmed by many researchers [22,25,26]

The study found that the risk of death was lower (*p* = 0.004, HR = 0.20) in patients struggling with obesity (BMI ≥ 30 kg/m^2^). Those patients were more likely to suffer from comorbidities such as HT and DM. They were also slightly younger ( $\overset{–}{X}$ = 67.57, SD = 11.64). Both obesity and malnutrition have a significant impact on the prevalence, the course, and the prognosis of HF. On the one hand, obesity is considered a risk factor for HF; on the other hand, it is a beneficial factor that is related to a lower risk of death—the “obesity paradox”. BMI itself is not a good indicator of obesity because it does not consider the exact body composition, i.e., amount of muscle, fat distribution, or water retention. However, due to its ease of use and accessibility, it is an integral part of the physical examination of HF patients [27,28]. However, obesity can lead to an abnormal clinical course [29]. Many studies confirm that obese patients have a better prognosis for short- and long-term survival [30,31,32]. However, does the obesity paradox apply to all patients with heart failure? Zamora et al. confirmed that obesity could act as a protective factor in patients with non-ischaemic heart failure. However, that phenomenon was not observed in patients with ischaemic heart failure [33]. The paradox phenomenon is controversial in the literature and clinical practice. Intensive treatment of obese patients may reduce mortality in this group. In some studies, obese patients were up to 10 years younger than patients with normal weight. Therefore, the physician may have decided to intensify the treatment, which might be the reason for lower mortality in this group [34]. Moreover, adipose tissue can be used as a nutrient when metabolism declines [35]. In many studies, the authors use BMI to assess whether a patient is overweight or obese. However, this index does not distinguish well between obesity phenotypes, thus the same patient with a BMI >30 may be an individual with an athletic physique, or sarcopenic obesity. To comprehensively assess the nutritional status of HF patients, a simultaneous assessment of diet and body composition is recommended. The bioelectrical impedance analysis (BIA) and the particularly dual-energy x-ray absorptiometry (DEXA) may be useful tools for assessing body composition. Those studies can help decide about appropriate nutritional and lifestyle interventions to preserve or increase muscle mass while decreasing fat mass [36]. As the phenomenon of obesity paradox continues to generate much uncertainty, this study may support further research in this area. However, it should be noted that patients’ nutritional status is an important factor affecting complications and a risk of long-term mortality. Efforts should be made to improve the nutritional status and lower the body weight, which has more potential benefits [37,38]. This is supported by the results of a meta-analysis conducted by the Global BMI Mortality Collaboration, based on 239 prospective studies from four continents. The aforementioned analysis showed that both overweight and obesity (regardless of their degree) were related to increased all-cause mortality [39]. However, that phenomenon requires scientific conclusions and further research.

### Study Limitation

The presented study had also some limitations. The first limitation was a small group of patients with an increased risk of malnutrition. Those patients constituted 4.3% of the study group (*N* = 1055), i.e., 45 individuals. In some cases, NRS scores and BMI results were not reported in medical records. The medical records also lacked information concerning patients’ prior treatment with, for example, lipid-lowering drugs. Patients were not screened for body composition analysis. Moreover, BMI results are not a reliable indicator for assessing overweight and obesity. The patients did not have their waist-to-hip circumference ratio (WHR) measured. The data concerning central (abdominal) obesity based on waist circumference was not reported either. The long-term survival of HF patients could not be assessed because of data restrictions due to the anonymity of medical records.

## 5. Conclusions

This study shows that the malnutrition status correlates with an increased risk of death during the hospitalisation of HF patients. Higher TC levels were related to a statistically significantly lower risk of death, which may indicate the “lipid paradox.” Higher BMI results were related to a statistically significantly lower risk of death, which may indicate the “obesity paradox.” Undoubtedly, those phenomena require further research.

## Figures and Tables

**Table 1:** Characteristics of the group with a comparison of survivors and non-survivors.

Variables	Total		Death					*p*-Value *
			No (998)			Yes (58)		
		*n*	*%*	*n*	%	*n*	%	
Sex (*n* = 1056)	M	704	66.7	671	67.2	33	56.9	0.10
BMI (*n* = 775)	<18.5	12	1.5	10	1.4	2	5.6	0.039
	18.5–24.9	203	26.2	194	26.3	9	25.0	
	25.0–29.9	264	34.1	247	33.4	17	47.2	
	≥30	296	38.2	288	39.0	8	22.2	
NRS (*n* = 1055)	<3	1010	95.7	964	96.7	46	79.3	<0.001
	≥3	45	4.3	33	3.3	12	20.7	
HF phenotype (*n* = 1056)	HFpEF	304	28.8	288	28.9	15	25.9	<0.001
	HFmrEF	130	12.3	125	12.5	16	27.6	
	HFrEF	551	52.2	529	53.0	5	8.6	
	No information	71	6.7	56	5.6	22	37.9	
NYHA (*n* = 986)	1	92	9.3	92	9.7	-	-	<0.001
	2	332	33.7	330	34.8	2	5.4	
	3	309	31.3	300	31.6	9	24.3	
	4	253	25.7	227	23.9	26	70.3	
CKD (*n* = 1056)	Yes	395	37.4	375	37.6	20	34.5	0.64
MI (*n* = 1056)	Yes	326	30.9	306	30.7	20	34.5	0.54
Type of MI (*n* = 1056)	No	730	69.1	692	69.3	38	65.5	0.90
	STEMI	90	8.5	84	8.4	6	10.3	
	NSTEMI	213	20.2	200	20.0	13	22.4	
	No information	23	2.2	22	2.2	1	1.7	
HT (*n* = 1056)	Yes	761	72.1	722	72.3	39	67.2	0.40
DM (*n* = 1056)	Yes	456	43.2	428	42.9	28	48.3	0.42
CS (*n* = 1056)	Yes	124	11.7	113	11.3	11	19.0	0.08
LDL (*n* = 1001)	<55 mg/dL	150	15.0	132	13.9	18	36.7	<0.001
	55 to <70 mg/dL	165	16.5	158	16.6	7	14.3	
	70 to <100 mg/dL	364	36.4	346	36.3	18	36.7	
	100 to <116 mg/dL	118	11.8	115	12.1	3	6.1	
	116 to <190 mg/dL	192	19.2	190	20.0	2	4.1	
	more than 190 mg/dL	12	1.2	11	1.2	1	2.0	
LDL (*n* = 1001)	≥70	686	68.5	662	69.5	24	49.0	0.003
TG (*n* = 1009)	<135	701	69.5	668	69.7	33	66.0	0.34
	135–200	207	20.5	198	20.6	9	18.0	
	>200	101	10.0	93	9.7	8	16.0	
HDL (*n* = 1005)	<40	522	51.9	485	50.7	37	75.5	0.001
	>40	483	48.1	471	49.3	12	24.5	
Variables	$\overset{–}{X}$	SD	$\overset{–}{X}$		SD	$\overset{–}{X}$	SD	*p*-Value **
Age (*n* = 1056)	69.73	12.92	69.27		12.95	77.72	9.20	<0.001
BMI [kg/m^2^] (*n* = 775)	28.97	6.20	29.07		6.22	27.01	5.44	0.052
Height [m] (*n* = 763)	169.33	9.13	169.43		9.11	167.31	9.44	0.17
Body weight [kg] (*n* = 761)	82.97	19.50	83.34		19.62	75.53	15.40	0.019
NT-proBNP [pg/mL] (*n* = 228)	8128.43	10,973.43	8135.63		11,085.30	7861.77	5944.59	0.95
BNP [pg/mL] (*n* = 775)	1046.69	1445.45	993.85		1424.94	1865.17	1528.66	<0.001
TG [mg/dL] (*n* = 1009)	124.36	67.25	124.25		67.30	126.40	66.98	0.83
LDL [mg/dL] (*n* = 1001)	89.38	36.24	90.32		36.18	71.16	32.66	<0.001
HDL [mg/dL] (*n* = 1005)	41.86	13.84	42.31		13.81	33.14	11.61	<0.001
TC [mg/dL] (*n* = 1010)	155.79	46.66	157.49		46.66	123.16	32.70	<0.001
CRP [mg/L] (*n* = 1023)	16.06	32.14	14.11		28.17	49.13	63.21	<0.001
Albumin [g/dL] (*n* = 278)	3.34	0.58	3.41		0.55	2.88	0.57	<0.001
Transferrin [g/L] (*n* = 261)	2.45	0.63	2.47		0.61	2.14	0.73	0.029
Lymphocytes [%] (*n* = 375)	20.32	9.94	21.22		9.68	11.80	8.21	<0.001
PCT [ng/mL] (*n* = 428)	0.82	3.59	0.62		3.00	2.39	6.44	0.001

Abbreviations: *n*, number of participants; M, males; $\overset{–}{X}$, mean; SD, standard deviation; *p*, level of significance; BMI, body mass index; NRS, nutritional risk screening; HF, heart failure; HFpEF, heart failure with preserved ejection fraction; HFmrEF, heart failure with mid-range ejection fraction; HFrEF, heart failure with reduced ejection fraction; NYHA, New York Heart Association classification; CKD, chronic kidney disease; MI, myocardial infarction; STEMI, ST-elevation myocardial infarction; NSTEMI, non-ST-elevation myocardial infarction; HT, arterial hypertension; DM, diabetes mellitus; CS, cerebral stroke; LDL, low-density lipoprotein; TG, triglycerides; HDL, high-density lipoprotein; NT-proBNP, N-terminal pro-B type natriuretic peptide; BNP, B-type natriuretic peptide; TC, total cholesterol; CRP, C-reactive protein; PCT, procalcitonin; * χ^2^ test; ** *t*-test.

**Table 2:** The comparison of assessed parameters (qualitative variables) with the ranges of BMI (WHO criteria) values.

Variables		BMI								*p*-Value *
		<18.5 *n* = 12		18.5–24.9 *n* = 203		25.0–29.9 *n* = 264		≥30 *n* = 296		
		*n*	%	*n*	%	*n*	%	*n*	%	
Sex	M	4	33.3	130	64.0	183	69.3	205	69.3	0.041
NRS	<3	10	83.3	189	93.1	253	95.8	290	98.0	0.009
	≥3	2	16.7	14	6.9	11	4.2	6	2.0	
HF phenotype	HFpEF	3	25.0	49	24.1	66	25.0	100	33.8	0.23
	HFmrEF	2	16.7	24	11.8	37	14.0	30	10.1	
	HFrEF	5	41.7	113	55.7	145	54.9	149	50.3	
	No information	2	16.7	17	8.4	16	6.1	17	5.7	
NYHA	1	2	20.0	25	13.6	27	10.8	20	7.1	0.26
	2	4	40.0	62	33.7	95	37.8	100	35.6	
	3	4	40.0	47	25.5	69	27.5	88	31.3	
	4	227	23.9	50	27.2	60	23.9	73	26.0	
CKD	Yes	5	41.7	72	35.5	91	34.5	129	43.6	0.11
MI	Yes	3	25.0	72	35.5	80	30.3	78	26.4	0.18
Type of MI	No	9	75.0	131	64.5	184	69.7	218	73.6	0.53
	STEMI	1	8.3	16	7.9	18	6.8	23	7.8	
	NSTEMI	2	16.7	50	24.6	53	20.1	51	17.2	
	No information	-	-	6	3.0	9	3.4	4	1.4	
HT	Yes	9	75.0	118	58.1	197	74.6	239	80.7	<0.001
DM	Yes	1	8.3	48	23.6	123	46.6	171	57.8	<0.001
CS	Yes	1	8.3	25	12.3	37	14.0	37	12.5	0.89
LDL	<55 mg/dL	-	-	17	8.9	40	15.6	48	17.1	0.09
	55 to <70 mg/dL	-	-	17	8.9	40	15.6	48	17.1	
	70 to <100 mg/dL	2	20.0	27	14.2	42	16.3	50	17.8	
	100 to <116 mg/dL	3	30.0	22	11.6	26	10.1	34	12.1	
	116 to <190 mg/dL	1	10.0	41	21.6	56	21.8	49	17.4	
	more than 190 mg/dL			6	3.2	2	0.8	1	0.4	
LDL	≥70	8	80.0	146	76.8	175	68.1	183	65.1	0.44
TG	<135	8	72.7	147	75.8	202	78.6	170	60.1	<0.001
	135–200	1	9.1	34	17.5	36	14.0	74	26.1	
	>200	2	18.2	13	6.7	19	7.4	39	13.8	
HDL	<40	5	50.0	76	40.0	119	46.1	178	62.9	<0.001
	>40	5	50.0	114	60.0	139	53.9	105	37.1	

Abbreviations: *n*, number of participants; M, males; *p*, level of significance; BMI, body mass index; NRS, nutritional risk screening; HF, heart failure; HFpEF, heart failure with preserved ejection fraction; HFmrEF, heart failure with mid-range ejection fraction; HFrEF, heart failure with reduced ejection fraction; NYHA, New York Heart Association classification; CKD, chronic kidney disease; MI, myocardial infarction; STEMI, ST-elevation myocardial infarction; NSTEMI, non-ST-elevation myocardial infarction; HT, arterial hypertension; DM, diabetes mellitus; CS, cerebral stroke; LDL, low-density lipoprotein; TG, triglycerides; HDL, high-density lipoprotein; * χ^2^ test.

**Table 3:** The comparison of assessed parameters (quantitative variables) with the ranges of BMI (WHO criteria) values.

Variables	BMI								*p*-Value **
	<18.5 *n* = 12		18.5–24.9 *n* = 203		25.0–29.9 *n* = 264		≥30 *n* = 296		
	$\overset{–}{X}$	SD	$\overset{–}{X}$	SD	$\overset{–}{X}$	SD	$\overset{–}{X}$	SD	
Age	73.42	9.95	69.55	14.40	70.81	12.10	67.57	11.64	0.013
NT-proBNP [pg/mL]	11,620.80	13,684.34	8447.83	9088.24	8832.30	11,774.43	4830.30	6711.13	0.047
BNP [pg/mL]	1405.63	1398.71	1410.75	1779.29	1033.11	1461.49	664.78	830.29	<0.001
TG [mg/dL]	110.00	71.80	113.43	55.74	117.62	71.61	137.72	70.51	<0.001
LDL [mg/dL]	90.70	22.58	96.95	40.09	89.43	37.27	86.28	33.75	0.021
HDL [mg/dL]	48.90	17.99	45.39	14.31	43.11	13.74	38.60	10.88	<0.001
TC [mg/dL]	154.73	41.11	164.60	50.59	155.90	47.33	152.25	41.52	0.039
CRP [mg/L]	22.86	45.93	14.65	28.77	13.88	29.28	16.86	31.54	0.54
Albumin [g/dL]	3.07	0.75	3.26	0.60	3.35	0.63	3.42	0.53	0.43
Transferrin [g/L]	2.56	0.42	2.23	0.58	2.62	0.64	2.47	0.68	0.022
Lymphocytes [%]	26.98	17.84	20.09	9.91	19.74	9.81	21.77	8.80	0.21
PCT [ng/mL]	1.42	3.60	0.91	3.75	1.25	5.49	0.38	1.50	0.38

Abbreviations: *n*, number of participants; $\overset{–}{X}$, mean; SD, standard deviation; *p*, level of significance; BMI, body mass index; NT-proBNP, N-terminal pro-B type natriuretic peptide; BNP, B-type natriuretic peptide; TG, triglycerides; LDL, low-density lipoprotein; HDL, high-density lipoprotein; TC, total cholesterol; CRP, C-reactive protein; PCT, procalcitonin; ** *t*-test.

**Table 4:** The comparison of assessed parameters (qualitative variables) with NRS scores.

Variables		NRS				*p*-Value *
		<3 (*n* = 1010)		≥3 (*n* = 45)		
		*n*	%	*n*	%	
Sex	M	677	67.03	26	57.78	0.20
BMI	<18.5	10	1.35	2	6.06	0.009
	18.5–24.9	189	25.47	14	42.42	
	25.0–29.9	253	34.10	11	33.33	
	≥30	290	39.08	6	18.18	
HF phenotype	HFpEF	290	28.71	14	31.11	0.017
	HFmrEF	126	12.48	4	8.89	
	HFrEF	532	52.67	19	42.22	
	No information	62	6.14	8	17.78	
NYHA	1	89	9.35	3	8.82	0.004
	2	328	34.45	4	11.76	
	3	299	31.41	10	29.41	
	4	236	24.79	17	50.00	
CKD	Yes	375	37.13	20	44.44	0.32
MI	Yes	309	30.59	16	35.56	0.48
Type of MI	No	701	69.4	29	64.4	0.46
	STEMI	83	8.2	6	13.3	
	NSTEMI	203	20.1	10	22.2	
	No information	23	2.3	29	64.4	
HT	Yes	730	72.28	30	66.67	0.41
DM	Yes	445	44.06	10	22.22	0.004
CS	Yes	118	11.68	6	13.33	0.74
LDL	<55 mg/dL	140	14.52	10	27.03	0.16
	55 to <70 mg/dL	161	16.70	4	10.81	
	70 to <100 mg/dL	348	36.10	16	43.24	
	100 to <116 mg/dL	114	11.83	4	10.81	
	116 to <190 mg/dL	189	19.61	3	8.11	
	more than 190 mg/dL	12	1.24			
LDL	≥70	663	68.78	23	62.16	0.39
TG	<135	672	69.35	29	72.50	0.63
	135–200	201	20.74	6	15.00	
	>200	96	9.91	5	12.50	
HDL	<40	495	51.08	27	75.00	0.005
	>40	474	48.92	9	25.00	

Abbreviations: *n*, number of participants; M, males; *p*, level of significance; BMI, body mass index; NRS, nutritional risk screening; HF, heart failure; HFpEF, heart failure with preserved ejection fraction; HFmrEF, heart failure with mid-range ejection fraction; HFrEF, heart failure with reduced ejection fraction; NYHA, New York Heart Association classification; CKD, chronic kidney disease; MI, myocardial infarction; STEMI, ST-elevation myocardial infarction; NSTEMI, non-ST-elevation myocardial infarction; HT, arterial hypertension; DM, diabetes mellitus; CS, cerebral stroke; LDL, low-density lipoprotein; TG, triglycerides; HDL, high-density lipoprotein; * χ^2^ test.

**Table 5:** The comparison of assessed parameters (quantitative variables) with NRS scores.

Variables	NRS				*p*-Value **
	<3 (*n* = 1010)		≥3 (*n* = 45)		
	$\overset{–}{X}$	SD	$\overset{–}{X}$	SD	
Age	69.36	12.83	78.02	12.33	<0.001
BMI [kg/m^2^]	29.11	6.20	25.83	5.39	0.003
Height [m]	169.52	9.03	164.87	10.39	0.006
Body weight [kg]	83.48	19.45	70.68	16.65	<0.001
NT-proBNP [pg/mL]	8012.61	10,979.46	13,293.70	10,443.66	0.29
BNP [pg/mL]	1021.53	1423.27	1612.36	1812.12	0.021
TG [mg/dL]	124.59	67.35	118.77	65.42	0.59
LDL [mg/dL]	89.86	36.35	76.95	31.25	0.033
HDL [mg/dL]	42.15	13.86	33.97	10.75	<0.001
TC [mg/dL]	156.78	46.66	130.97	39.62	0.001
CRP [mg/L]	15.30	31.37	32.90	43.11	<0.001
Albumin [g/dL]	3.34	0.58	3.26	0.55	0.49
Transferrin [g/L]	2.46	0.63	2.16	0.57	0.08
Lymphocytes [%]	20.74	9.85	13.61	9.03	0.001
PCT [ng/mL]	0.74	3.44	1.89	5.09	0.09

Abbreviations: *n*, number of participants; $\overset{–}{X}$, mean; SD, standard deviation; *p*, level of significance; BMI, body mass index; NT-proBNP, N-terminal pro-B type natriuretic peptide; BNP, B-type natriuretic peptide; TG, triglycerides; LDL, low-density lipoprotein; HDL, high-density lipoprotein; TC, total cholesterol; CRP, C-reactive protein; PCT, procalcitonin; ** *t*-test.

**Table 6:** Survival time.

		Survival Time [Days]
**Percentiles**	25 percentiles (lower quartile)	26.0
	50 percentiles (median)	39.3
	75 percentiles (upper quartile)	66.7

**Table 7:** Descriptive statistics for survival time, number of deaths, and survival rate according to BMI results, NRS scores, and LDL scores.

		Descriptive Statistics				
		Me	$\overset{–}{X}$	SD	*N*—Death	*N*—Survivors
BMI	<18.5	12.5	13.8	10.7	2	10
	18.5–24.9	7.0	8.2	8.7	9	194
	25.0–29.9	6.0	7.9	7.2	17	247
	≥30	6.0	8.7	9.4	8	288
NRS	<3	6.0	8.0	7.7	46	964
	≥3	9.0	13.6	14.0	12	33
LDL	<55 mg/dL	7.0	8.8	8.7	18	132
	55 to <70 mg/dL	7.0	7.9	6.5	7	158
	70 to <100 mg/dL	6.0	8.6	9.4	18	346
	100 to <116 mg/dL	6.0	7.5	5.8	3	115
	116 to <190 mg/dL	6.0	7.6	7.2	2	190
	more than 190 mg/dL	4.5	4.8	2.8	1	11

Abbreviations: *n*, number of participants; Me, median; $\overset{–}{X}$, mean; SD, standard deviation; BMI, body mass index; NRS, nutritional risk screening; LDL, low-density lipoprotein.

**Table 8:** The assessment of the influence of variables on mortality—a Cox proportional hazards regression, single model.

		*p*-Value	HR	95%CI HR (Lower)	95%CI HR (Upper)
Sex (*n* = 1056)	M	0.283	0.75	0.45	1.27
BMI (*n* = 775)	18.5–24.9	Ref.			
	<18.5	0.486	3.26	0.49	21.75
	25.0–29.9	0.151	1.60	0.70	3.66
	≥30	0.028	0.51	0.19	1.32
NRS (*n* = 1055)	<3	Ref.			
	≥3	0.014	2.31	1.19	4.49
HF phenotype (*n* = 1056)	HFpEF	Ref.			
	HFmrEF	0.000	4.69	2.02	10.91
	HFrEF	0.376	0.91	0.33	2.50
	No information	0.067	0.84	0.44	1.61
NYHA (*n* = 986)	1	Ref.			
	2	0.988	0.00	0.00	
	3	0.991	0.18	0.04	0.76
	4	0.988	0.37	0.17	0.82
MI (*n* = 1056)	Yes	0.343	1.30	0.75	2.26
CKD (*n* = 1056)	Yes	0.321	0.76	0.44	1.31
Type of MI (*n* = 1056)	No	Ref.			
	STEMI	0.451	1.72	0.28	10.46
	NSTEMI	0.849	1.18	0.62	2.23
	No information	0.990	1.25	0.17	9.16
HT (*n* = 1056)	Yes	0.506	0.83	0.48	1.44
DM (*n* = 1056)	Yes	0.714	1.10	0.65	1.86
CS (*n* = 1056)	Yes	0.249	1.48	0.76	2.87
LDL (*n* = 1001)	<55 mg/dL	Ref.			
	55 to <70 mg/dL	0.826	0.65	0.11	3.82
	70 to <100 mg/dL	0.413	0.34	0.06	2.13
	100 to <116 mg/dL	0.386	0.06	0.01	0.48
	116 to <190 mg/dL	0.009	0.10	0.02	0.45
	more than 190 mg/dL	0.038	3.20	0.41	25.22
LDL (*n* = 1001)	≥70	0.003	0.42	0.24	0.75
TG (*n* = 1009)	<135	Ref.			
	135–200	0.656	1.90	0.89	4.05
	>200	0.130	2.53	1.16	5.52
HDL (*n* = 1005)	<40	Ref.			
	>40	0.110	0.58	0.30	1.13
Variables					
Age (*n* = 1056)		0.000	1.05	1.02	1.07
BMI [kg/m^2^] (*n* = 775)		0.048	0.94	0.89	1.00
Height [m] (*n* = 763)		0.435	0.99	0.95	1.02
Body weight [kg] (*n* = 761)		0.019	0.98	0.96	1.00
NT-proBNP [pg/mL] (*n* = 228)		0.779	1.00	1.00	1.00
BNP [pg/mL] (*n* = 775)		0.006	1.00	1.00	1.00
TG [mg/dL] (*n* = 1009)		0.050	1.00	1.00	1.01
LDL [mg/dL] (*n* = 1001)		0.002	0.98	0.97	0.99
HDL [mg/dL] (*n* = 1005)		0.001	0.95	0.93	0.98
TC [mg/dL] (*n* = 1010)		0.000	0.98	0.98	0.99
CRP [mg/L] (*n* = 1023)		0.000	1.01	1.01	1.01
Albumin [g/dL] (*n* = 278)		0.000	0.34	0.19	0.62
Transferrin [g/L] (*n* = 261)		0.126	0.56	0.27	1.18
Lymphocytes [%] (*n* = 375)		0.000	0.92	0.88	0.96
PCT [ng/mL] (*n* = 428)		0.003	1.07	1.02	1.12

Abbreviations: *n*, number of participants; M, males; HR, hazard ratio; CI, confidence interval; *p*, level of significance; BMI, body mass index; NRS, nutritional risk screening; HF, heart failure; HFpEF, heart failure with preserved ejection fraction; HFmrEF, heart failure with mid-range ejection fraction; HFrEF, heart failure with reduced ejection fraction; NYHA, New York Heart Association classification; CKD, chronic kidney disease; MI, myocardial infarction; STEMI, ST-elevation myocardial infarction; NSTEMI, non-ST-elevation myocardial infarction; HT, arterial hypertension; DM, diabetes mellitus; CS, cerebral stroke; LDL, low-density lipoprotein; TG, triglycerides; HDL, high-density lipoprotein; NT-proBNP, N-terminal pro-B type natriuretic peptide; BNP, B-type natriuretic peptide; TC, total cholesterol; CRP, C-reactive protein; PCT, procalcitonin.

**Table 9:** The assessment of the influence of variables on mortality (Cox proportional hazards regression, multivariate model).

		Beta	Standard Error	Chi-Square	*p*-Value	HR	95% CI HR (Lower)	95%CI HR (Upper)
TC		−0.02	0.01	7.79	0.005	0.98	0.97	0.99
BMI	Underweight	1.24	0.80	2.369	0.12	12.30	0.80	189.92
	Overweight	0.04	0.39	0.009	0.93	0.87	0.29	2.65
	Obesity	−1.45	0.50	8.447	0.004	0.20	0.05	0.79
TG	135–200	0.65	0.46	2.007	0.16	6.15	1.69	22.40
	>200	0.52	0.55	0.899	0.34	5.42	1.03	28.36

Abbreviations: HR, hazard ratio; CI, confidence interval; *p*, level of significance; BMI, body mass index; TG, triglycerides; TC, total cholesterol.

## Data Availability

The data will be available by contacting the corresponding author.
